# Integration of RNA-seq and ATAC-seq identifies muscle-regulated hub genes in cattle

**DOI:** 10.3389/fvets.2022.925590

**Published:** 2022-08-11

**Authors:** Jianfang Wang, Bingzhi Li, Xinran Yang, Chengcheng Liang, Sayed Haidar Abbas Raza, Yueting Pan, Ke Zhang, Linsen Zan

**Affiliations:** ^1^College of Animal Science and Technology, Northwest A&F University, Xianyang, China; ^2^National Beef Cattle Improvement Center, Northwest A&F University, Xianyang, China

**Keywords:** muscle, RNA-seq, WGCNA, hub genes, ATAC-seq, transcription factor

## Abstract

As the main product of livestock, muscle itself plays an irreplaceable role in maintaining animal body movement and regulating metabolism. Therefore, it is of great significance to explore its growth, development and regeneration to improve the meat yield and quality of livestock. In this study, we attempted to use RNA-seq and ATAC-seq techniques to identify differentially expressed genes (DEGs) specifically expressed in bovine skeletal muscle as potential candidates for studying the regulatory mechanisms of muscle development. Microarray data from 8 tissue samples were selected from the GEO database for analysis. First, we obtained gene modules related to each tissue through WGCNA analysis. Through Gene Ontology (GO) functional annotation, the module of lightyellow (ME_lightyellow_) was closely related to muscle development, and 213 hub genes were screened as follow-up research targets. Further, the difference analysis showed that, except for PREB, all other candidate hub genes were up-regulated (muscle group vs. other-group). ATAC-seq analysis showed that muscle-specific accessible chromatin regions were mainly located in promoter of genes related to muscle structure development (GO:0061061), muscle cell development (GO:0055001) and muscle system process (GO:0003012), which were involved in cAMP, CGMP-PKG, MAPK, and other signaling pathways. Next, we integrated the results of RNA-seq and ATAC-seq analysis, and 54 of the 212 candidate hub genes were identified as key regulatory genes in skeletal muscle development. Finally, through motif analysis, 22 of the 54 key genes were found to be potential target genes of transcription factor MEF2C. Including *CAPN3, ACTN2, MB, MYOM3, SRL, CKM, ALPK3, MAP3K20, UBE2G1, NEURL2, CAND2, DOT1L, HRC, MAMSTR, FSD2, LRRC2, LSMEM1, SLC29A2, FHL3, KLHL41, ATXN7L2*, and *PDRG1*. This provides a potential reference for studying the molecular mechanism of skeletal muscle development in mammals.

## Introduction

The focus of the adjustment of the new agricultural industrial structure is to develop high-quality and efficient stock husbandry. Compared with pig and poultry breeding, cattle breeding can greatly improve the utilization of straw resources and reduce environmental pollution, so it plays an increasingly important role in the development of animal husbandry. As the largest tissue of cattle organisms, the growth, development and genetic characteristics of skeletal muscle affect and even determine the meat production performance of cattle (1). Many factors are closely related to muscle tenderness and affect meat product quality, such as intramuscular fat content, muscle fiber size, and intramuscular protein content and types (2–5). The growth and development of skeletal muscle is an extremely complex biological process, which is co-regulated by a variety of regulatory factors.

Transcription factors (TFs) are critical in muscle growth and development. Members of the myogenic regulatory factor (MRF) family are typical inducers of skeletal muscle development, including the early myogenic regulatory factors (MYOD, MYF5, MYF6, MRF4, et al.) and the late differentiation marker gene (MyoG) (6). Myocyte enhancer Factor-2 (MEF2) family (MEF2a, MEF2b, MEF2c, and MEF2d) is a kind of key transcription factor discovered after MyoD, which controls the expression of myogenic genes (7). The N-terminal of MEF2 contains a highly conserved MADS-Box domain and its adjacent MEF2 domain. The MADS-Box domain mediates protein dimerization, while the MEF2 domain influences its binding affinity with DNA and its interaction with cofactors (7–9). It has been reported that MEF2s was involved in regulating the proliferation and differentiation of myocyte mainly through participating in Ca^2+^-calmodulin-dependent protein kinases CaMK-histone deacetylases HDACs, Calconneurin, MAPK and other signaling pathways (10, 11). In addition, with the rise of RNA-seq technology, a large number of genes related to muscle development have been discovered successively, but the signal regulatory networks between genes and genes, transcription factors and target genes, and epistasis and genes are still poorly understood. Moreover, we focused more on finding the few regulators that play a dominant role in the complex regulatory network and exploring their relevant regulatory mechanisms *in vivo*.

As chromatin is the carrier of genes, the activation and silencing of genes will inevitably cause the remodeling of chromatin structure. ATAC-seq is a newly developed technique to study the open regions of genomic chromatin, which helps to elucidate the regulatory mechanisms of genes (12). In recent years, a large number of researchers began to explore the relationship between chromatin open region and gene expression, including domestic animals. A study found a group of highly functionally conserved gene regulatory elements in different tissues by comparing the genomes of three domestic animals (Gallus Galla, Sus Scrofa, Bos Taurus) to human and mouse genomes (13). Furthermore, by comparing chromatin accessibility in muscle, liver and hypothalamus of Bos indicus cattle, learned that MEF2 is the main regulator of muscle-specific open chromatin region (14). Combined with previous studies, we intend to further explore the key regulatory genes involved in skeletal muscle development by comparing the sequencing data of muscle and other tissues, and explore the transcription factors that may be regulated, which will provide some methodological guidance and reference value for the screening and functional research of core genes.

## Materials and methods

### Data acquisition

**Figure 1:**
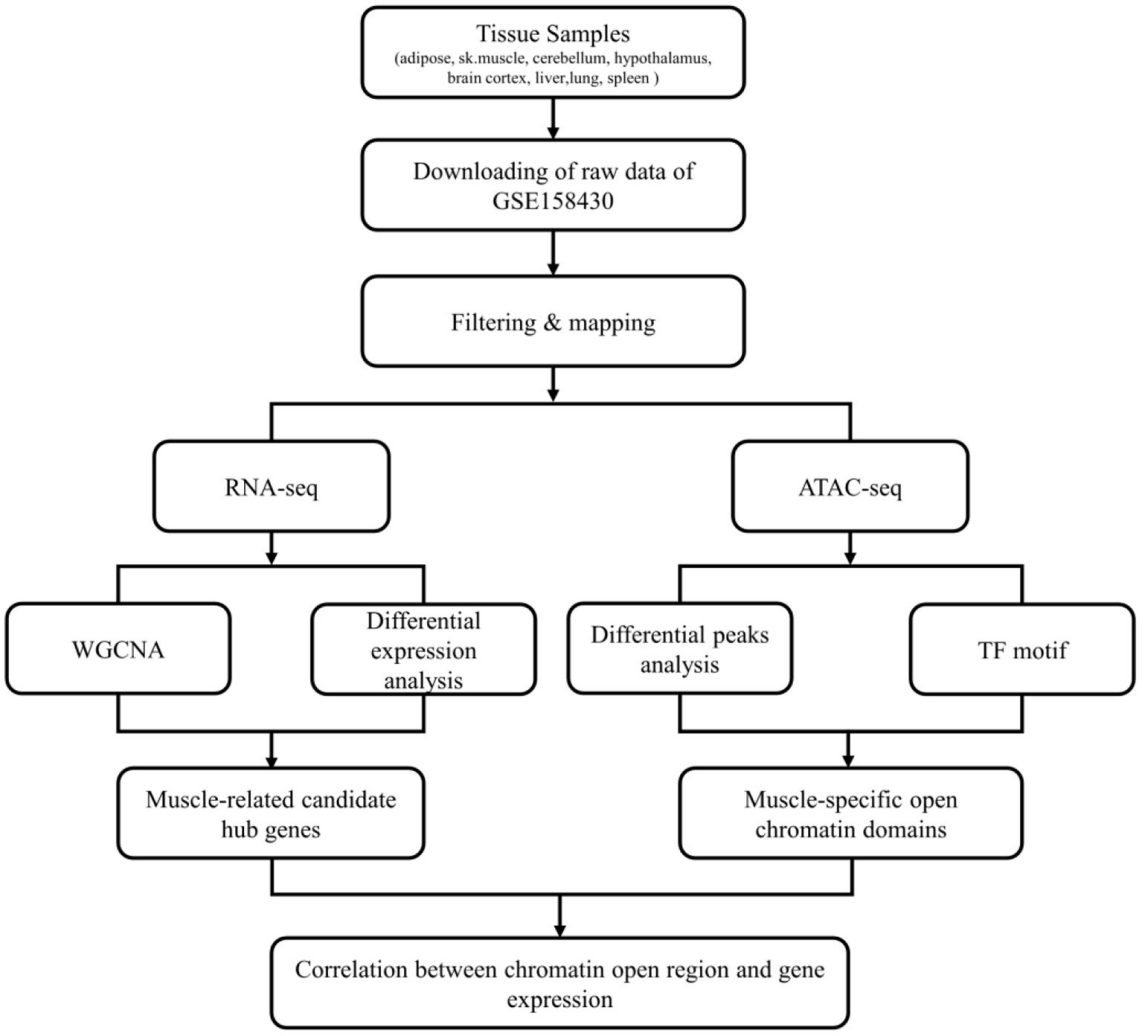
The flow chart of whole analysis.

The workflow of this study was shown in Figure 1. The data used in this study was from GSE158430 of the GEO database (GEO Accession viewer (nih.gov) (13). GSE158430 is a SuperSeries and has three SubSeries (GSE158412, GSE158414, and GSE158416). GSE158412 dataset contains RNA sequencing data from 8 different tissues (liver, lung, spleen, skeletal muscle, subcutaneous adipose, cerebellum, brain cortex, and hypothalamus) of cattle, pigs, and chickens, with two biological replicates. GSE158414 dataset includes ATAT-seq data from different tissues of cattle and pigs with two biological replicates (except cerebellum tissue in cattle). GSE158416 was CTCF ChIP-seq in 8 different tissues of the above livestock species, which also has two biological replicates. We only downloaded the SRA format data of cattle in GSE158412 and GSE158414 for subsequent analysis (see Supplementary Table 1 for details). The SRA format was then converted to fastq format using sratools' fastq-dump command (15).

### Expression matrix pre-processing

To obtain high-quality clean reads, Trimmomatic (v0.36) (16) and FastQC (http://www.bioinformatics.babraham.ac.uk/projects/fastqc/) were used to filter and evaluate the quality of sequencing reads. Clean reads were mapped to cattle (*Bos taurus*) reference genome from Ensembl Genome Browser 104 (http://asia.ensembl.org/index.html) using hisat2 (v2.1.0) (17). SAMtools (v1.7) was used to convert the sam files obtained from mapping to bam files (18). Two important values representing gene expression were tallied: reads count was quantified with featureCounts (v1.6.0) (19), The FPKM (fragments per kilobase of transcript per million fragments mapped) were calculated using StringTie (v2.1.2) (20). Further, the genes were annotated through Ensembl Genome Browser 104 database, and protein-coding genes (PCGs) were screened for WGCNA and Differently Expression analysis (21).

### Weighted gene coexpression network analysis

The top 75% of PCGs with the highest median absolute deviation (MAD) were constructed the co-expression network with the value of log_2_(FPKM+1) using WGCNA package (v1.70) from Bioconductor in RStudio (v1.2) (http://www.rstudio.org), an integrated development environment for R (v4.0.2). Then, the weighted adjacency matrix of 16 tissue samples was constructed by the formula of the adjacency matrix: amn = |cor(mn)|β (amn, adjacency between m and n; cor(mn), Pearson's correlation between m and n; and β, soft-power threshold) (22). Subsequently, the adjacency matrix was transformed into a topological overlap matrix (TOM) to quantitatively describe the similarity in nodes by comparing the weighted correlation between two nodes and other nodes. Next, hierarchical clustering was used to identify modules according to the TOM matrix with the minModuleSize 100. Similar modules (>75%) were merged (abline = 0.25).

### Identification of sample significant modules and hub genes

Module-sample associations were estimated according to TOM (|cor| > 0.5, *P* < 0.01). The same module of genes is generally considered to have a higher topological overlap similarity. For each expression profile, gene significance (GS, the correlation between expression profile and each sample) and module membership [MM, the correlation between the module eigengene (ME) and the gene expression profile] were used to identify the Module-sample correlation. Finally, hub genes screened with MM > 0.8 and GS > 0.6 with weighted *P*-value (*P*.weighted) < 0.01.

### RNA differential expression analysis

The expression matrix of whole genes was used for further analysis of gene expression differences among different samples by DEseq2 in R software (23). False Discovery Rate (FDR) was got by adjusting the *P*-value. Genes with fold change > 2 and false discovery rate (FDR) < 0.05 were set as the threshold to be DEGs.

### ATAC-seq analysis

First, FastQC was used to detect data quality. The clean reads were obtained by removing low-quality fragments using Fastp (v0.19.4) (24). BWA (Burrows-Wheeler Alignment) (v0.7.17) was used to map clean reads to cattle (Bos taurus) reference genome. Genrich (v0.6.1) was used to calling peaks for each sample (https://github.com/jsh58/Genrich). The different peaks were identified using DiffBind (https://bioconductor.org/packages/release/bioc/html/DiffBind.html) (v2.16.2) with the default parameters (25). ChIPseeeker (v1.24.0) (26) was used to annotate the differential peaks according to the Ensembl Genome Browser 104 (http://asia.ensembl.org/index.html) and org.Bt.eg.db (https://bioconductor.org/packages/release/data/annotation/html/org.Bt.eg.db.html). During the genome-wide annotation of peaks, the genome-wide functional regions were divided into promoter, dowstream, exon, intron and distal intergenic regions. The closest gene to each peak can be obtained according to the distance of each binding site to TSS, and their specific distribution of binding sites on the genome can be found out. This study considered the profile of peaks at their ± 3 kb of the transcription start site (TSS) as promoter genes or targets. The motifs were examined using the Multiple EM for Motif Elicitation (MEME) suite (https://meme-suite.org/meme/doc/meme-chip.html?man_type=web). Then, the Motif database scanning algorithm TOMTOM was used to predict transcription factors (TFs) (27). ATACseqQC (v1.12.5) was used for quality detection and motif footprint analysis (28). FIMO was used to scan binding locations of TFs in the promoter regions of DEGs (upstream 2,000 bp and downstream 100 bp) (29).

### Gene ontology and kyoto encyclopedia of genes and genomes analysis

To explore the potential biological roles of module genes, Gene Ontology (GO) functional annotation for multiple gene lists was analyzed using clusterProfile (30). And the GO analysis for the Single-gene list was carried out by g:Profiler (https://biit.cs.ut.ee/gprofiler/gost) with the default parameters (31). KEGG pathway analyses were performed using KOBAS (32). We used a significant threshold *P*-value adjusted by Benjamini and Hochberg of 0.05 for KEGG to control the false discovery rate (FDR) and used Fisher's Exact Test as the statistical test method.

### Real-time quantitative PCR

The total RNA from bovine tissues or cells was extracted by Trizol reagent kit (Invitrogen, Carlsbad, CA, USA) and then PrimeScript™ RT reagent Kit (Takara, Dalian, China) was used to synthesize cDNA. Real-time quantitative PCR (qRT-PCR) was used to measure the mRNA expression levels (Supplementary Table 2) with TB Green® Premix Ex Taq™ II (Takara, Dalian, China) and CFX Connect Real-Time PCR Detection System. The amplification procedure was as follows: 95°C, 30 s; followed by 40 cycles (95°C, 5 s; 60°C, 30 s) and stored at 4°. Quantitative results were analyzed by the 2^−ΔΔCt^ method for relative expression. All samples contained 3 biological replicates and 3 technical replicates. All data are expressed as Mean ± SD. The unpaired two-tailed Student's *t*-test was used for statistical analysis. In this study, lowercase letters a-g were used to indicate significance (*P* < 0.05).

## Results

### Construction of co-expression network and identification of muscle-related modules

**Figure 2:**
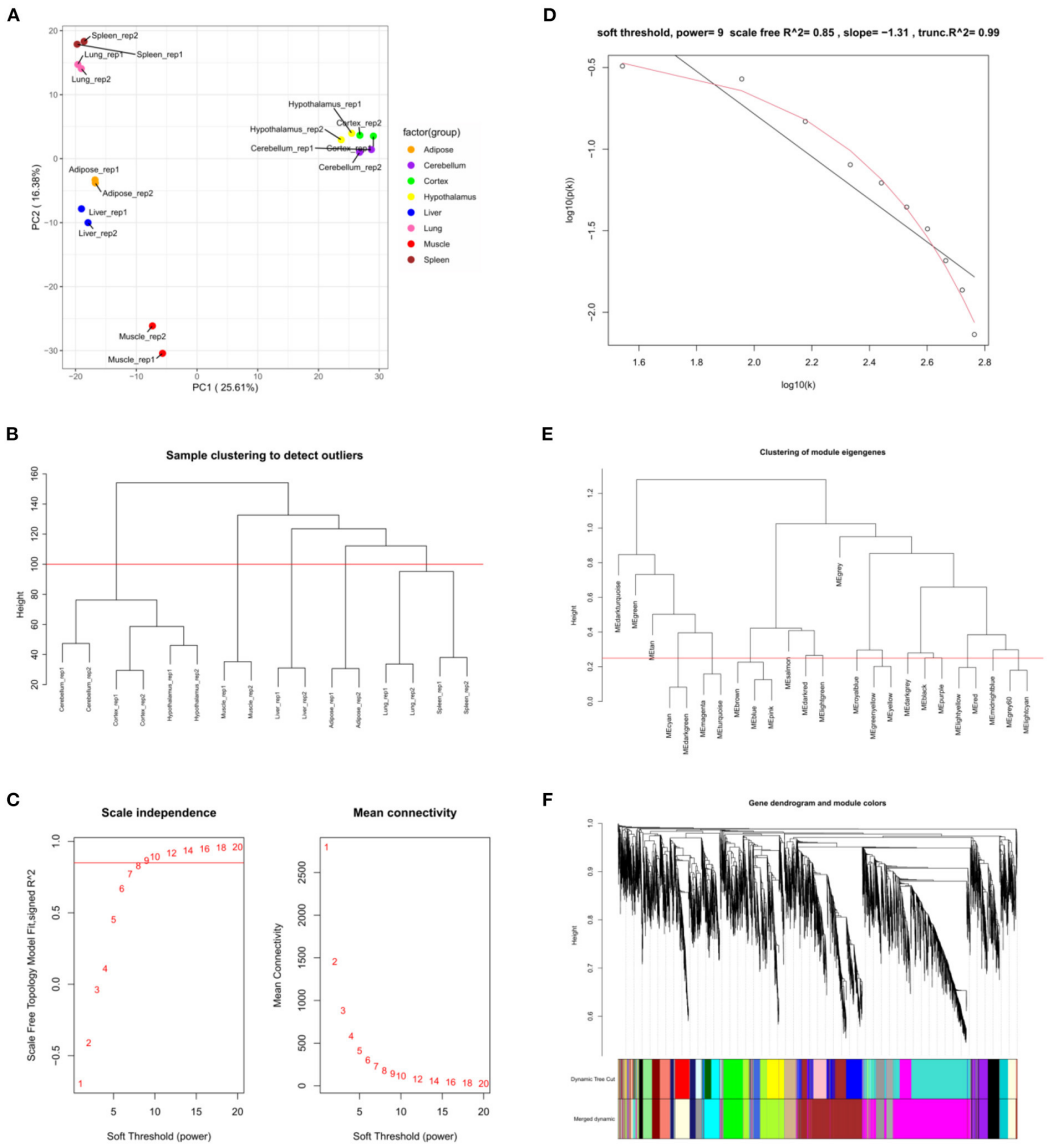
Weighted co-expression network analysis. **(A)** Principal component analysis of all samples. **(B)** Sample clustering tree. The red line represents the outlier elimination reference line; **(C)** Soft-threshold powers (β) filtering analysis. The mean connectivity (y-axis) for various soft-threshold powers (x-axis) was showed on the left, and the mean connectivity (y-axis) of different soft-thresholding power (x-axis) was showed on the right; **(D)** Checking the scale-free topology when β = 9. X-axis: the log10 (network connectivity), y-axis: log10 (the corresponding frequency distribution). The distribution approximates a straight line, which is called the approximately scale-free topology; **(E)** The clusting of module eigengenes. The red line represents the reference line for similar module merging; **(F)** The cluster dendrogram of modules.

**Figure 3:**
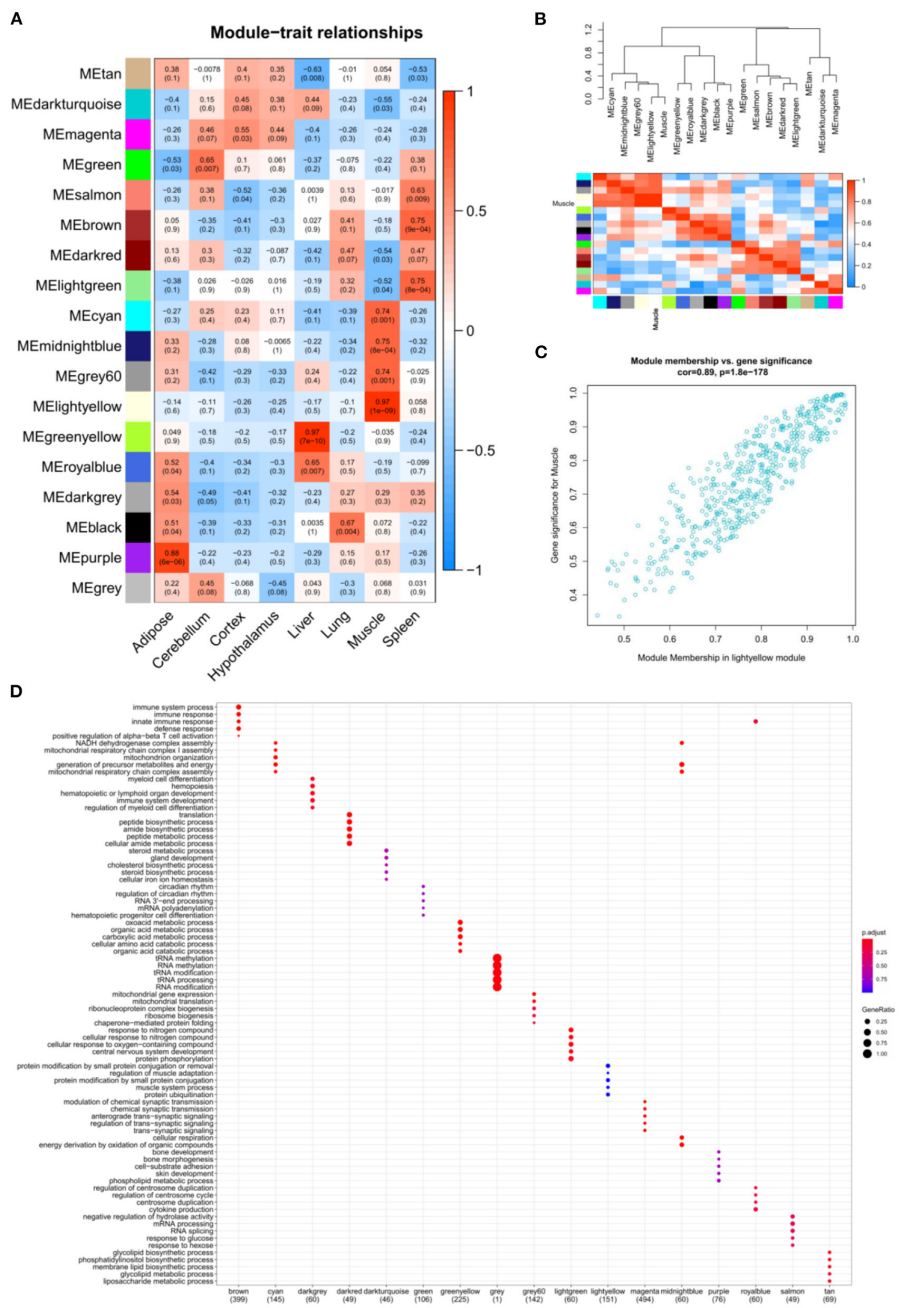
The relation analysis between modules and samples. **(A)** Heatmap of the module-sample correlation. Significance (*P*-value) was marked in parentheses. The color bar represented the magnitude of the correlation. Red: positive-correlation, blue: negative-correlation; **(B)** The adjacency heatmap of eigengene, including the module clustering tree (top) and the corresponding module clustering heatmap (below). The color bar represented the magnitude of the correlation. Red: positive-correlation, blue: negative-correlation; **(C)** The scatterplot of GS and MM in MElightyellow module; **(D)** The top 5 significant GO-BP analysis form each module eigengenes.

GSE158412 dataset contains 16 tissue samples of cattle. Before the analysis, we first tested the credibility of biological repetition of the samples by principal component analysis, and the result showed that there was clustering between 2 repeats of 8 tissues (Figure 2A). All samples could be used for analysis. After processing raw data, the gene expression matrix for 10,034 protein-coding genes was obtained. Among them, 7,525 genes (the top 75% of the median absolute deviation) were used to construct the weighted co-expression network. The Pearson's correlation coefficient (PCC) was performed to construct the sample clustering tree (Figure 2B). Then, WGCNA's pickSoftThreshold function was selected to estimate the best value of power (β). In this study, the best value of power was estimated as 9 (β = 9) (scale-free R^2^ = 0.85) (Figure 2C). The scale-free topology was performed to test the reliability of β = 9 (Figure 2D). Furthermore, the dynamic hierarchical tree cutting algorithm was used to detect common expression modules, and then similar modules (minimum height was set to 0.25) were combined (Figure 2E). Finally, a total of 18 modules were identified (Figures 2E,F). The eigengenes contained in each module was provided in Supplementary Table 3. The correlation between modules and samples was shown in Figure 3A. We found that four modules were highly correlated with muscle tissue, namely ME_cyan_ (cor = 0.74, *P* = 0.001), ME_midnightblue_ (cor = 0.75, *P* = 8E-04), ME_grey60_ (cor = 0.74, *P* = 0.001) and ME_lightyellow_ (cor = 0.97, *P* = 1E-09). The adjacency heatmap of the relationship for each model was shown in Figure 3B. In addition, GS and MM were highly correlated (cor = 0.89, *P* =1.8E-178), suggesting that genes that are highly correlated with the characteristics of muscle samples are also the most important (central) elements of modules associated with that sample (Figure 3C). Through roughly performing GO annotation on eigengenes of each module, we found that ME module with the highest correlation to muscle development, including regulation of muscle adaptation and muscle system process, etc. (Figure 3D). Therefore, this study finally extracted the ME_lightyellow_ module for further analysis. In ME_lightyellow_, 213 out of 519 genes (~41%) met the screening conditions of hub genes (MM > 0.8, GS > 0.6, *P.weighted* < 0.01) in Supplementary Table 4.

### Screening of candidate hub genes with differential expression

**Figure 4:**
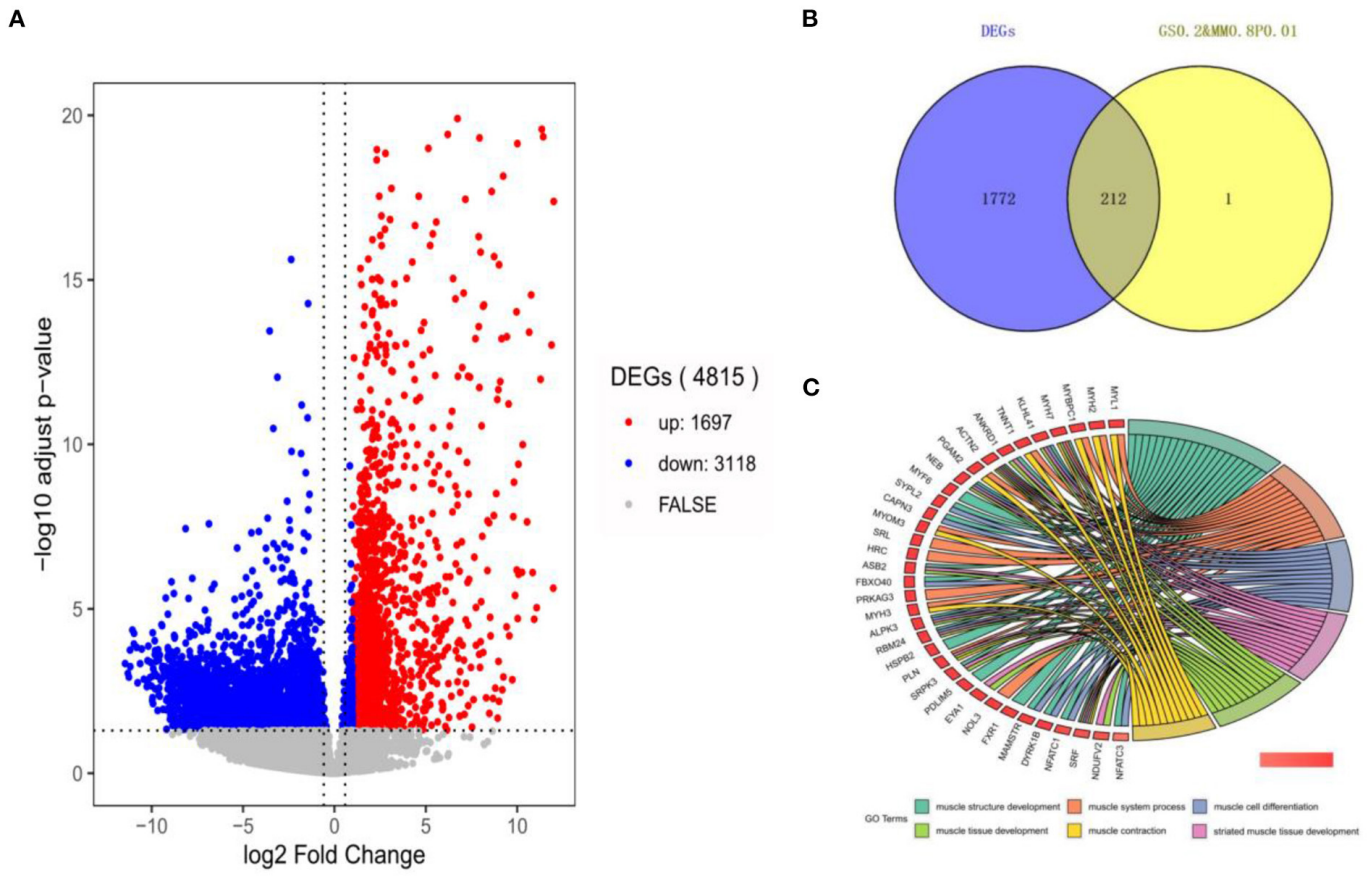
The analysis of DEGs by RNA-seq. **(A)** The volcano-plot of DEGs; **(B)** The overlap-plot of DEGs and candidate hub genes in MElightyellow; **(C)** The top 6 GO terms with the high significance of 212 candidate hub genes.

In this section, 10,034 protein-coding genes were analyzed to identify muscle-specific DEGs. The result showed that a total of 4,815 DEGs were found, including 1,697 DEGs (up-regulated) in the muscle group and 3,118 DEGs (down-regulated) in the other-group. The analysis profile of DEGs was visualized in Figure 4A. The top 10 up-regulated DEGs were *CKM, TNNC1, MYOT, SLN, ACTA1, MYL1, TNNC2, MYLPF, MYH2*, and *MYL2*, and the top 10 down-regulated DEGs were *GAD2, CLVS2, NRXN3, CELF3, ST8SIA3, GRM5, GRIN1, SCRT1, GRIN2A*, and *MYT1* (Table 1; Supplementary Table 5). GO and KEGG results are provided in the supplied materials (Supplementary Tables 6–9). Further analysis found that 212 candidate hub genes (no include PREB) related to muscle development were finally screened by integration difference analysis and WGCNA analysis (Figure 4B). GO analysis was performed on these 212 candidate hub genes with different changes, and the result showed that these genes were also enriched in terms related to muscle development (Figure 4C; Supplementary Table 10), demonstrating the feasibility of integrating differential analysis and WGCNA to obtain muscle-related hub genes.

**Table 1:** Top 10 up- and down-regulated expressed genes.

**Genes**	**Description**	**log_**2**_FoldChange**	***P*value**	***P*_adj_**
CKM	Creatine kinase, M-type	13.45451834	1.41E-209	5.72E-206
TNNC1	Troponin C1	13.45401812	3.51E-128	5.70E-125
MYOT	Myotilin	13.44382896	1.09E-205	2.53E-202
SLN	Sarcolipin	13.39986882	3.53E-86	2.39E-83
ACTA1	Actin alpha 1	13.37281374	1.54E-206	5.00E-203
MYL1	Myosin light chain 1	13.32452561	0	0
TNNC2	Troponin C2	13.31786432	3.10E-134	5.60E-131
MYLPF	Myosin light chain, phosphorylatable	13.31718808	3.82E-80	2.30E-77
MYH2	Myosin heavy chain 2	13.3004601	5.69E-97	4.40E-94
MYL2	Myosin light chain 2	13.28565102	2.31E-110	2.51E-107
GAD2	Glutamate decarboxylase 2	−24.51019661	3.21E-15	2.56E-13
CLVS2	Clavesin 2	−24.45531689	3.28E-15	2.60E-13
NRXN3	Neurexin 3	−24.05692778	3.25E-14	2.34E-12
CELF3	Neurexin 3	−23.93374376	2.20E-14	1.61E-12
ST8SIA3	CUGBP Elav-like family member 3	−23.74276415	2.62E-15	2.11E-13
GRM5	ST8 alpha-N-acetyl-neuraminide alpha-2,8-sialyltransferase 3	−23.70823989	1.38E-11	7.20E-10
GRIN1	Glutamate metabotropic receptor 5	−23.6503701	1.11E-10	5.15E-09
SCRT1	Glutamate ionotropic receptor NMDA type subunit 1	−23.5113897	4.87E-12	2.75E-10
GRIN2A	Scratch family transcriptional repressor 1	−23.46027351	5.45E-12	3.06E-10
MYT1	Glutamate ionotropic receptor NMDA type subunit 2A	−23.25118573	3.92E-15	3.09E-13

### The landscape of genomic chromatin accessibility

To analyze the influence of chromatin open region on the differential expression of the candidate hub genes, the ATAC-seq technique was applied for further analysis. The raw data of GSE158414 were re-analyzed to obtain the open chromatin region of each sample. Simply, clean reads were first obtained by removing low-quality reads and adaptors, and then mapped to the reference genome (*Bos taurus*). Genrich (https://github.com/jsh58/Genrich) is a new software for analyzing ATAC-seq. It can remove mitochondrial reads, PCR repeats, multiple mapping reads, and multiple biological repeats with just one command. So, Genrich software was used to call peak in this study. The number of peaks for each sample was counted and stored in Supplementary Table 11. To determine whether the sequencing fragments were the open regions of the genome, all samples were selected for the analysis of the distribution of fragment lengths. In all samples analyzed, the 100 bp single-nucleosome fragments in leftmost were found, rather than single or multiple-nucleosome fragments, which indicates that these peaks were from open regions of the genome (Supplementary Figure 1). All results showed the good quality of ATAC-seq. The TSS of genes with active transcription is often open, so the signal profile near TSS is another important factor to identify the quality of ATAC-seq.

**Figure 5:**
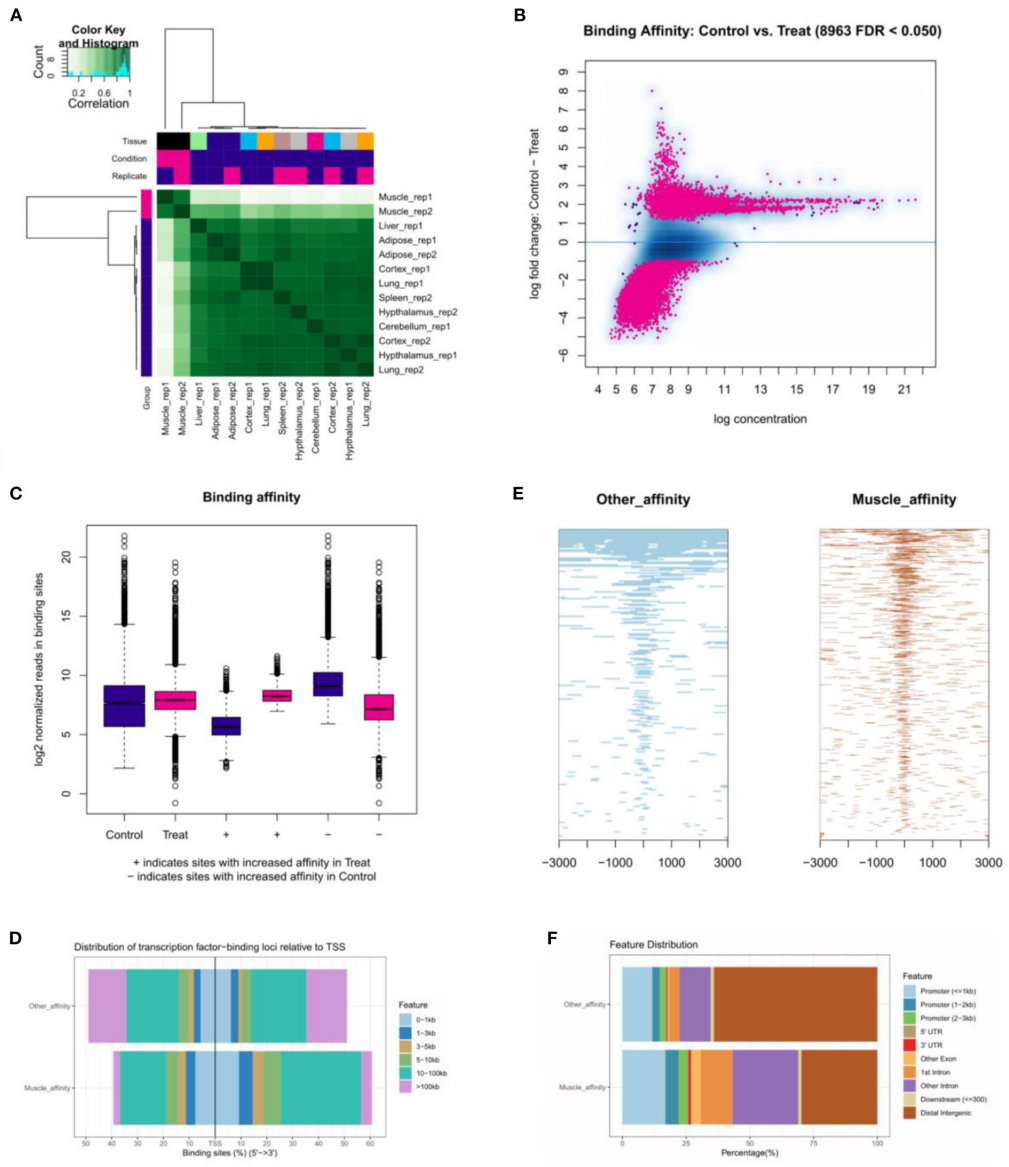
The landscape of genomic chromatin accessibility. **(A)** The correlative heatmap of tissues; **(B)** MA plot of difference peaks analysis; **(C)** The box-plot of binding affinity; **(D)** The location distribution of different peaks distance TSS; **(E)** The heatmap of peaks around TSS; **(F)** The distribution of function regions of different peaks.

The correlative heatmap of tissues was shown significant differences between muscles and other tissues (Figure 5A). Next, through analyzing the differential accessible peaks, 8,963 different peaks were obtained (FDR < 0.05), including 4,564 muscle-affinitive peaks and 4,398 other-affinitive peaks in muscle vs. other. MA-plot of different peaks was shown in Figure 5B, and box-plot of binding affinity was shown in Figure 5C. The annotation result showed that most of the peaks were 10–100 kb away from TSS (Figure 5D). The distribution of these binding sites tends to be at the 3 'end of TSS, which is consistent with the results of previous studies (33). In addition, Figure 5E showed the peak enriched heatmap near TSS. Many peaks enriched near the TSS (±3 kb) of genes, indicating that transcription factors are likely to bind to these chromatin open regions.

**Figure 6:**
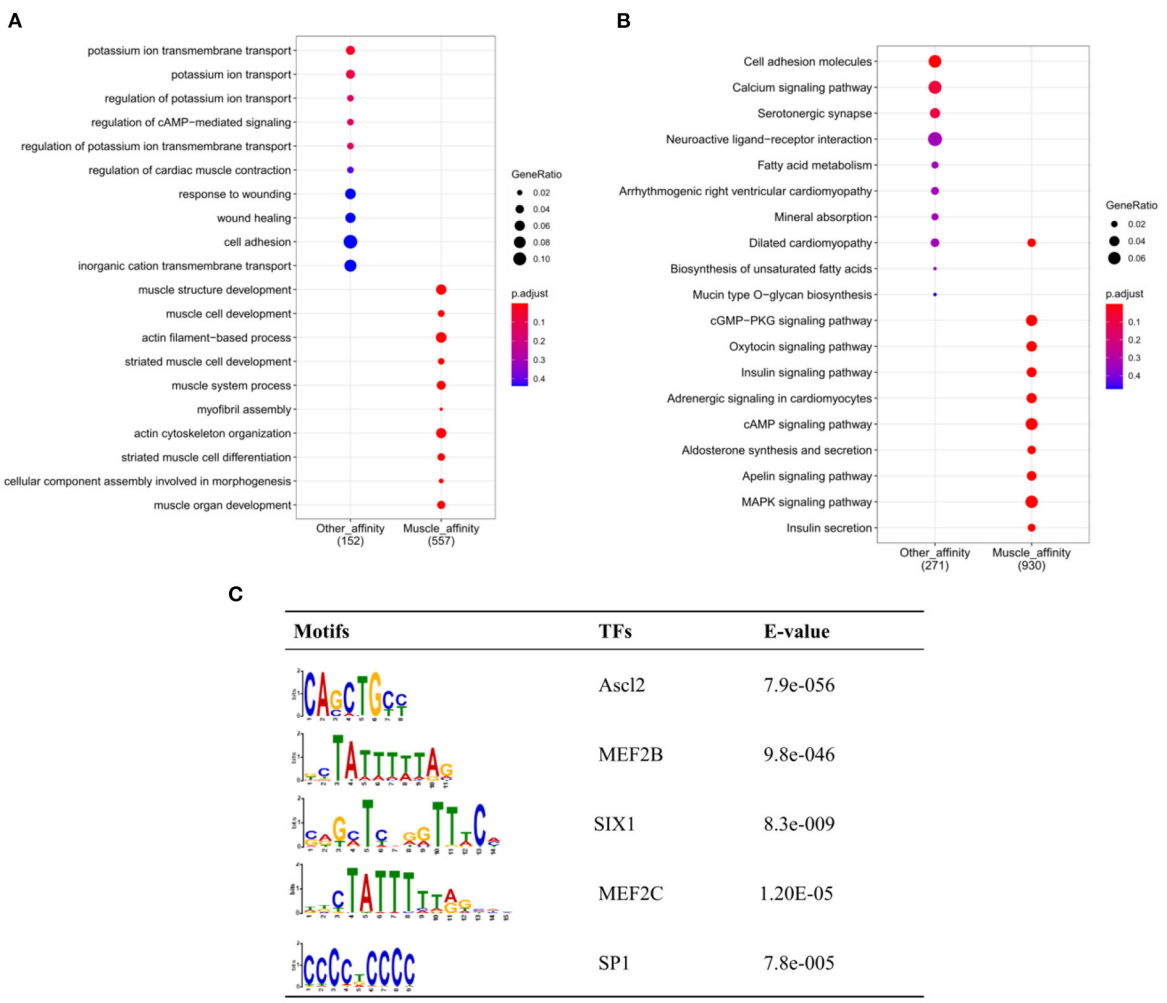
Genomic enrichment analysis. **(A)** GO-BP analysis of different peaks; **(B)** Enrichment analysis of KEGG pathway; **(C)** The top 5 predicted binding known motif in the open chromatin region.

To further explore the functional differences between muscle-affinitive peaks and other-affinitive peaks, we performed gene functional region annotation, and found that compared with other-affinitive peaks, muscle-affinitive peaks were enriched in promoter and exon regions significantly. Enrichment of distal intergenic was significantly reduced (Figure 5F). Peaks located in the promoter (called promoter-peaks) are critical to gene expression, so we extracted the genes in the promoter region for GO and KEGG analysis. The GO-BP analysis indicated that muscle-affinitive genes were related to muscle structure development (GO:0061061), muscle cell development (GO:0055001), muscle system process (GO:0003012), and so on (Figure 6A). KEGG analysis found muscle-affinitive genes were involved in cAMP, cGMP-PKG, MAPK, and other related functional pathways (Figure 6B). In addition, we found that several key TFs of muscle development were found to be significantly enriched at these ATAC-seq peaks (Figure 6C), suggesting muscle development may be related to the binding of transcription factors to open regions of chromatin.

### Integration analysis of ATAC-seq and RNA-seq

**Figure 7:**
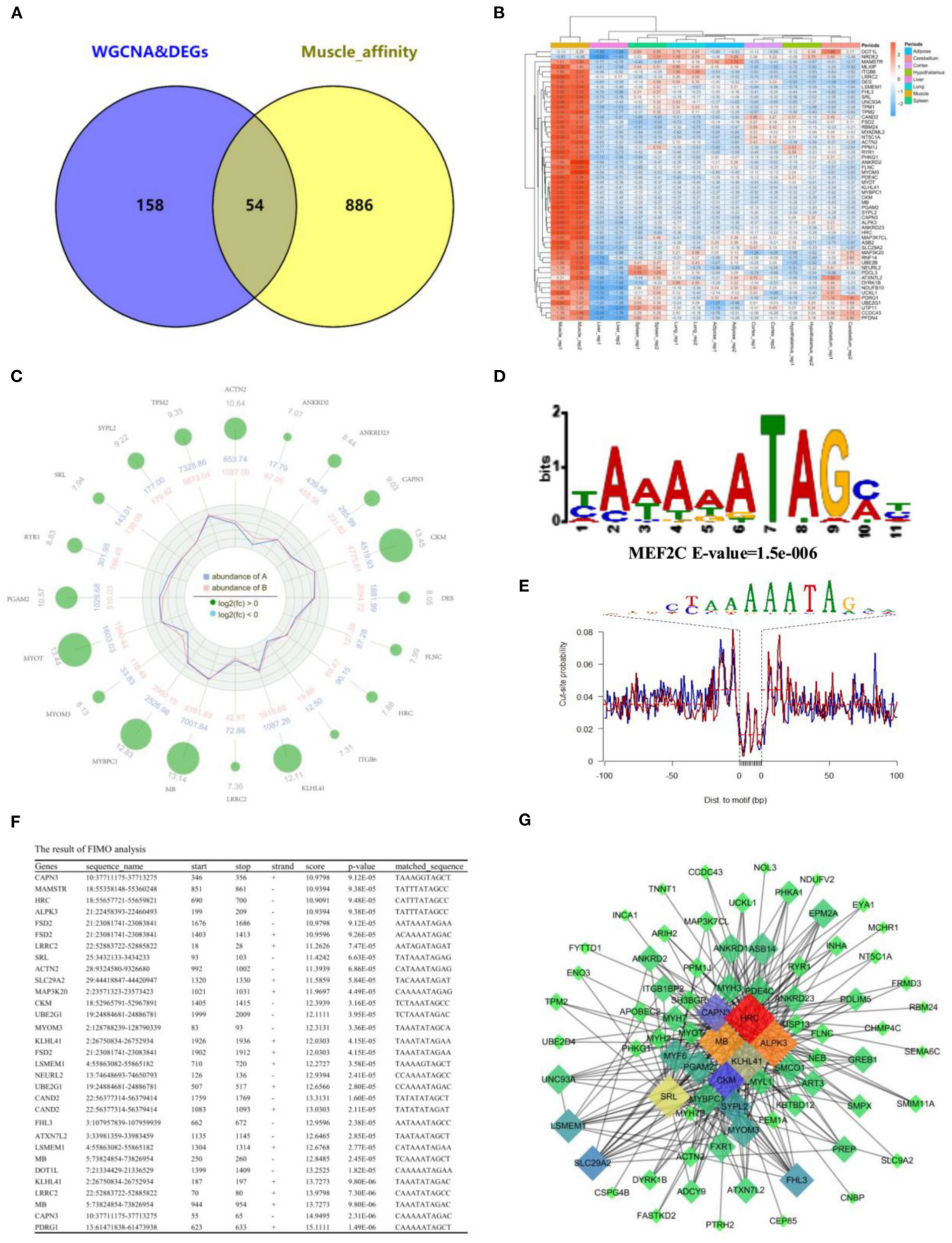
Integration of RNA-Seq and ATAC-Seq. **(A)** Venn plot of ATAC-seq and RNA-seq, and 54 common genes were found; **(B)** Heatmap plot of common genes; **(C)** Radar plot of the top 20 common genes; **(D)** Motif enrichment analysis of common genes around open chromatin regions. MEF2C was found to be significantly enriched; **(E)** The motif footprint analysis of MEF2C; **(F)** Motif Scanning of MEF2C in promoter region of common gene using FIMO; **(G)** The subregulatory network of 22 predicted MEF2C potential target genes in the ME_light_ module was derived from WGCNA analysis.

To further determine the relationship between gene expression and open chromatin regions, we overlapped the analysis results from RNA-seq and ATAC-seq, respectively. Fifty-four common muscle-regulated genes were found (Figure 7A). The expression heatmap of these muscle-regulated genes was shown in Figure 7B, and we found these genes have a significant difference between muscle and other tissues. The top 20 genes with high differential expression were shown in Figure 7C, including *MYOT, MYOM3*, Mymyon (*MB*), Kelch Like Family Member 41 (*KLHL41*), Actinin Alpha 2 (*ACTN2*), and *CAPN3* and other muscle-specific expression genes. Again, TF binding analysis was performed on the open chromatin regions corresponding to these 54 genes. The promoter-peaks of 44 of the 54 genes (~81.48%) were enriched by MEF2C which was the only transcription factor discovered (Figure 7D). The motif footprint analysis of MEF2C was showed in Figure 7E. In addition, we extracted the promoter regions of the 54 genes (upstream 2,000 bp and downstream 100 bp) using bedtools, and analyzed whether the MEF2C promoter is binding in these genes. Twenty-two of 54 hub genes (50%) were found to have MEF2C binding sites (*P* < 0.0001) (Figure 7F). By visually selecting the interaction between these genes and the target genes in Me_lightylow_ with the value of weight > 0.3, MB, HRC, KLHL41, SRL, and ALPK3 was found that occupy important positions in the regulatory network (Figure 7G). They are muscle-specific and muscle-regulated genes, suggesting that the combination of ATAC-seq and RNA-seq make our screening more accurate.

### Validate the expression of hub genes

**Figure 8:**
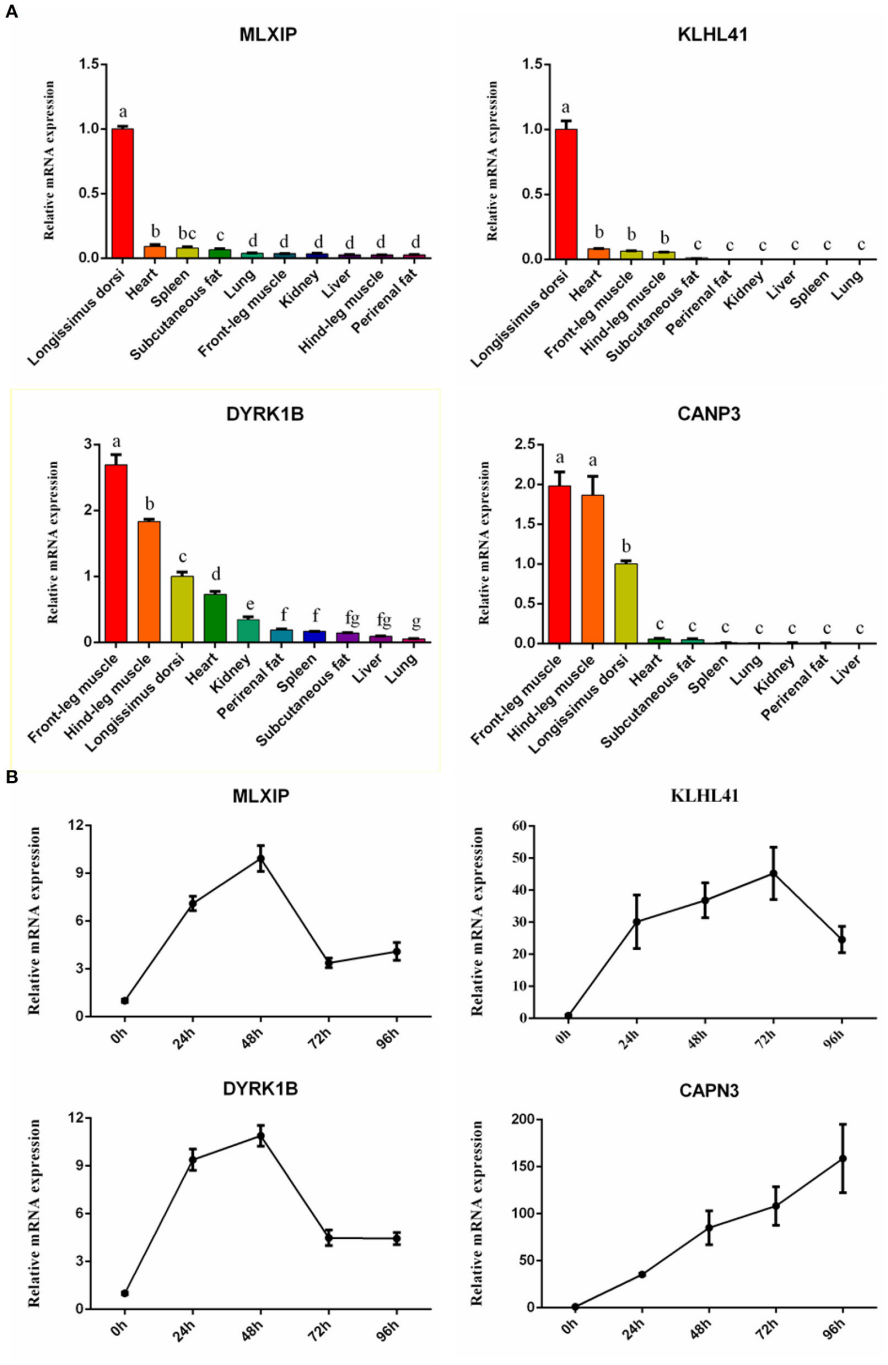
The relative expression of hub genes by qRT-PCR. **(A)** The bar plots represented tissue expression profiles. **(B)** The line charts represented myogenic cell differentiation expression profiles.

To further confirm our results, 4 high-expression hub genes in muscle, including *CAPN3* and *KLHL41* targeted by MEF2C, and dual specificity tyrosine phosphorylation regulated kinase 1B (*DYRK1B*) and MLX interacting protein (*MLXIP*), were randomly selected for qRT-PCR. The results showed that the expression of these four genes in muscle tissue was significantly higher than that in other tissues, which was highly consistent with the results of RNA-seq (Figure 8A). In addition, we quantified the expression of these four genes in the process of myocyte differentiation, and found that their expression levels increased with the increase of the degree of differentiation of muscle cells, suggesting these genes might also play a crucial role in the differentiation (Figure 8B). Therefore, the screening of muscle-affinitive hub genes through ATAC-seq and RNA-seq can be further studied in the future on the molecular mechanism of muscle proliferation and differentiation.

## Discussion

Different from other tissues, the development of muscle, the main product of beef cattle, directly affects the economic value of beef. To improve beef yield from a genetic perspective, many novel techniques have been applied to explore the mechanism of muscle development in cattle. Among them, bulk RNA-seq has been widely promoted as a routine bulk screening technique for muscle-related genes (34, 35). However, the differentially expressed genes screened by it are often in thousands, and it is still difficult to obtain the core regulatory genes from them. In contrast, weighted gene co-expression network analysis (WGCNA) could be used to characterize correlation patterns between genes and samples (21). Candidate hub genes were identified according to the endogeneity of gene-sets and the association between gene-sets and samples (36). Compared to focusing only on differentially expressed genes, WGCNA is able to use genome-wide information to obtain our gene sets of interest, which not only narrows the screening scope but also makes the analysis more accurate. This technique has been applied extensively to plants, humans, mice, poultry, and livestock (36–41).

In this study, we analyzed the association of genes with different tissues using WGCNA, and obtained 4 module gene-sets with high positive correlation with muscle tissue. Combined with the results of GO functional annotation, we finally targeted ME_lightyellow_ as the study target, from which we screened 213 candidate hub genes (GS > 0.6, MM > 0.8) involved in the positive regulation of muscle development. Compared with other tissues, 212 of 213 (99.53%) candidate hub genes were up-regulated expression in muscle [log2(FC) > 1, FDR < 0.05], which not only verified the robustness of WGCNA analysis, but also provided a reliable collection for the subsequent study.

In eukaryotes, DNA and histones are tightly bound and stored in nucleosomes (chromatin), chromatin structure, nucleosome location and histone modifications affect DNA transcription. Chromatin accessibility is closely related to the binding of regulatory elements or transcription factors, which are particularly important for gene activation and repression. ATAC-seq, an innovative technique for detecting chromatin accessibility, has been increasingly applied to determine the mechanisms of gene expression regulation with RNA-seq (42). In this study, we found that the chromatin open regions of muscle tissue were significantly different from other tissues. The results of functional annotation revealed that muscle-affinitive peaks were more enriched in exons and promoter regions compared to other tissues, suggesting that these chromatin-open regions are associated with gene expression regulation. In addition, we performed GO and KEGG analysis of DEGs around promoter-peaks and found that muscle-affinitive peaks were enriched on the hub genes specifically expressed by muscle as expected. It was involved in the regulation of cAMP, cGMP-PKG, and MAPK signaling pathways. Similar results were obtained in early studies on mouse tissue-specific genes (43). Among them, MAPK signaling pathway plays a crucial role in in the formation, regeneration, movement and injury repair of skeletal muscle (44–46).

Combined with ATAC-seq, 54 genes with high expression in muscle were identified as muscle-regulated hub genes. To further analyze the regulatory mechanism of these 54 hub genes in the development of skeletal muscle, we conducted motif analysis of the chromatin open region fragments. Twenty-two of the 54 genes were predicted to be regulated by MEF2C. This is consistent with earlier tissue analysis in mice (47). MEF2C is a member of the myocyte enhancer factor 2 family, which itself lacks myogenic activity and was initially considered to regulate muscle development by activating the transcriptional activity of bHLH myogen proteins (such as MyoD and MyoG) (48–50). Herein, we analyzed the possible MEF2C involved in downstream target genes, we broadly classified them into 3 major categories: 1. The muscle-specific genes, including *CAPN3, ACTN2, MB, KLHL41*, Cullin associated and neddylation dissociated 2 (*CAND2*), Myomesin 3 (*MYOM3*) and sarcalumenin (*SRL*); 2. The genes of kinases and epigenetic enzymes, including creatine kinase, M-Type (*CKM*), alpha kinase 3 (*ALPK3*), mitogen-activated protein kinase kinase kinase 20 (*MAP3K20*), ubiquitin conjugating enzyme E2 G1 (*UBE2G1*), Neuralized E3 Ubiquitin protein ligase 2 (NEURL2) and DOT1 like Histone lysine methyltransferase (*DOT1L*); 3. other genes with transcriptional regulation functions, including Histidine rich calcium binding protein (*HRC*), MEF2 activating motif and SAP domain containing transcriptional regulator (*MAMSTR*), fibronectin type III and SPRY domain containing 2 (*FSD2*), leucine rich repeat containing 2 (*LRRC2*), leucine rich single-pass membrane protein 1 (*LSMEM1*), solute carrier family 29 member 2 (*SLC29A2*), four and a half LIM domains 3 (*FHL3*), Ataxin 7 like 2 (*ATXN7L2*) and p53 and DNA damage regulated 1 (*PDRG1*).

In this study, some well-known myogenic genes have been reported to be involved in myoblast proliferation and differentiation dependent on the transcriptional regulation of MEF2, such as *CAPN3, MB, Myomesin*, and *CKM* (51–54). More interestingly, HRC, a direct target gene of MEF2, encodes histidine-rich calcium-binding protein (HRCBP) involved in the regulation of muscle development mainly in the sarcoplasmic reticulum of cardiac and skeletal muscles and in the calcium bodies of smooth muscle (55, 56). *MASTR*, a coactivator of MEF2, is involved in the regulation of myoblast proliferation and differentiation by encoding a cofactor that stimulates MEF2C (57, 58). These studies confirm the reliability of the results of this study. Other genes have not been reported to interact directly with MEF2C, but many have been reported to be involved in the regulation of muscle. It has been reported that *ACTN2* and its family gene *ACTN3* encode myosin α-actin-2 and α-actin-3 proteins, respectively, which constitute the Z-line in mammalian skeletal muscle fibers (59). SRL is a Ca^2+^-binding protein localized in the sarcoplasmic reticulum (SR) affecting skeletal muscle movement (60). MAP3K20 is involved in the regulation of the JNK/MAPK signaling pathway, which plays a role in muscle development and regeneration (61). UBE2G1 encodes an E2 ubiquitin-coupled enzyme that functions mainly in the ubiquitin-proteasome system, which is involved in muscle degradation and regeneration (62). NEURL2 is capable of encoding proteins involved in the regulation of myogenic fiber organization and ubiquitin-mediated degradation of β-linked proteins during myogenesis (63). *DOT1L* is a key epigenetic gene that mediates H3K79me2 modifications involved in cardiomyocyte differentiation (64). *LRRC2* is a member of the LRRC family and its role in epigenetic modifications in skeletal muscle has also been reported (65). *KLHL41* is predominantly expressed in skeletal muscle and is essential for the maintenance of skeletal muscle integrity and myogenic fiber formation (66, 67). *FHL3* has been reported to regulate myogenic differentiation and muscle-specific gene expression by acting as a transcriptional co-activator or co-repressor (68). *FSD2* is highly expressed in cardiac and skeletal muscle as a candidate gene affecting sarcomere traits in animals (69). *CAND2* and is a muscle-specific expression gene mediated by mTORC1 that affects cardiac remodeling but not skeletal muscle (70). *ALPK3* has been reported to be closely associated with familial cardiomyopathy, but its role in skeletal muscle has not been reported (71). In this study, *ALPK3* was located at the core of the interaction network of candidate genes with *MB, HRC, SRL*, and *KLHL41*, so we speculate that it also is irreplaceable for skeletal development. *LSMEM1, SLC29A2, ATXN7L2*, and *PDRG1* have not been directly reported to be associated with muscle development. In conclusion, these genes are worthy of our attention and their potential regulatory role with transcription factors and their epistatic modifications in muscle development is of great interest to explore.

Notably, this study used other tissues as the background and only contested the analysis of genes with positive regulation with muscle. In fact, there are many more information that can be mined to be further analyzed and verified, such as other tissue-related genes, functional analysis of other modular genes, and joint analysis with other epistatic data.

## Data availability statement

The datasets presented in this study can be found in online repositories. The names of the repository/repositories and accession number(s) can be found in the article/Supplementary material.

## Ethics statement

The animal study was reviewed and approved by Ethics Committee of Northwest A&F University.

## Author contributions

JW, BL, and LZ conceived and designed the experiments. JW wrote the manuscript. XY, CL, SR, YP, and KZ contributed sample collection and reagents preparation, and analyzed the data. JW, BL, XY, CL, SR, YP, KZ, and LZ revised the manuscript. All authors reviewed the manuscript. All authors contributed to the article and approved the submitted version.

## Funding

This work was supported by the Natural Science Foundation of China (31972994), Key Research and Development Program of Ningxia Province (2019BEF02004), National Beef and Yak Industrial Technology System (CARS-37), Key Research and Development Program of Shaanxi Province (2022NY-050 and 2022ZDLNY01-01), Transformation Project of Shaanxi Province (NYKJ-2018-LY09) and Special Project for the Central Government to Guide Local Science and Technology Development (2060404-51301).

## Conflict of interest

The authors declare that the research was conducted in the absence of any commercial or financial relationships that could be construed as a potential conflict of interest.

## Publisher's note

All claims expressed in this article are solely those of the authors and do not necessarily represent those of their affiliated organizations, or those of the publisher, the editors and the reviewers. Any product that may be evaluated in this article, or claim that may be made by its manufacturer, is not guaranteed or endorsed by the publisher.
