# Supplementary figures and images for: Integration of RNA-seq and ATAC-seq identifies muscle-regulated hub genes in cattle
Source: Front Vet Sci. 2022 Aug 11;9:925590. doi: 10.3389/fvets.2022.925590 (PMC9404375; doi:10.3389/fvets.2022.925590)

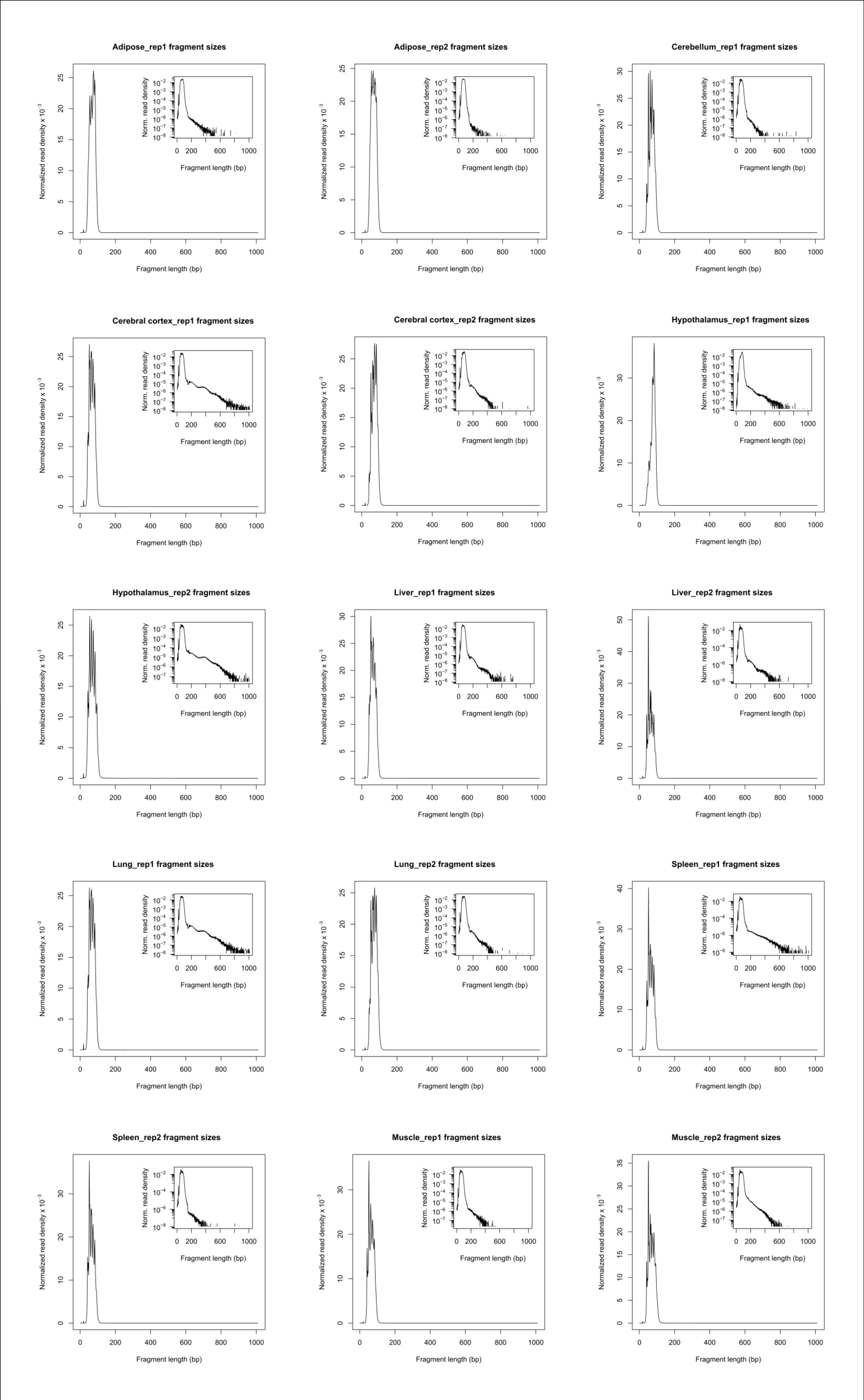

Supplement: Supplementary file 12 [file Image_1.JPEG]
